# Immunological Effects of Dimethyldioctadecylammonium Bromide and Saponin as Adjuvants for Outer Membrane Vesicles from *Neisseria meningitidis*

**DOI:** 10.3390/diseases10030046

**Published:** 2022-07-19

**Authors:** Victor Araujo Correa, Amanda Izeli Portilho, Elizabeth De Gaspari

**Affiliations:** 1 Immunology Center, Adolfo Lutz Institute, Av. Dr. Arnaldo, 355, 11th Floor, Room 1116, Cerqueira César, São Paulo 01246-902, SP, Brazil; viktor-araujo@hotmail.com (V.A.C.); a.izeliportilho@usp.br (A.I.P.); 2 Graduate Program Interunits in Biotechnology, Biomedical Sciences Institute, São Paulo University, Av. Prof. Lineu Prestes, 2415, ICB Hall III, Cidade Universitária, São Paulo 05508-900, SP, Brazil

**Keywords:** outer membrane vesicles, aluminium hydroxide, dimethyldioctadecylammonium bromide, saponin, prime booster immunization

## Abstract

The meningococcal disease is a global health threat, but is preventable through vaccination. Adjuvants improve meningococcal vaccines and are able to trigger different aspects of the immune response. The present work evaluated the immune response of mice against *Neisseria meningitidis* outer membrane vesicles (OMV) complexed with the adjuvants aluminium hydroxide (AH), via subcutaneous route; and dimethyldioctadecylammonium bromide (DDA) or Saponin (Sap), via intranasal/subcutaneous routes. ELISA demonstrated that all adjuvants increased IgG titers after the booster dose, remaining elevated for 18 months. Additionally, adjuvants increased the avidity of the antibodies and the bactericidal titer: OMVs alone were bactericidal until 1:4 dilution but, when adjuvanted by Alum, DDA or Sap, it increased to 1/32. DDA and Sap increased all IgG isotypes, while AH improved IgG1 and IgG2a levels. Thus, Sap led to the recognition of more proteins in Immunoblot, followed by DDA and AH. Sap and AH induced higher IL-4 and IL-17 release, respectively. The use of adjuvants improved both cellular and humoral immune response, however, each adjuvant contributed to particular parameters. This demonstrates the importance of studying different adjuvant options and their suitability to stimulate different immune mechanisms, modulating the immune response.

## 1. Introduction

*Neisseria meningitidis* is a Gram-negative diplococcus, responsible for meningococcal disease, manifested by septicemia and/or meningitis. It is lethal in 10% of cases, and 30–50% of the survivors acquire morbidities [1]. The chemical composition of the capsular polysaccharide is responsible for meningococcus classification in serogroups [2,3].

Polysaccharide vaccines conjugated with proteins have been used to prevent the disease caused by serogroups A, C, W and Y [4]. The capsular polysaccharide of serogroup B, composed of the α,2-8 linked sialic acid is similar to the polysialic acid found in neuronal human cells. Therefore, a polysaccharide B vaccine would be poorly immunogenic and could lead to autoimmunity [3]. The outer membrane vesicles (OMV), which present several subcapsular-protein antigens started to be used to develop meningococcal B vaccines, which succeeded in controlling serogroup B outbreaks [5,6]. More recently, recombinant outer membrane proteins, selected to improve cross-reactivity, were associated with OMV from an epidemic strain to develop the MenB-4C (Bexsero^®^) vaccine [4,7].

The current meningococcal vaccines are administrated intramuscularly. This route can elicit a robust systemic humoral response, but provides a weak T cell-mediated immunity and lacks mucosal protection [8]. On the other hand, when vaccines are administrated by heterologous prime-booster strategy, combining mucosal and parenteral routes, a rapid and wide biodistribution of the antigen was observed, with a capacity to induce mucosal and systemic cellular and humoral responses [9]. Studies pointed to promising results combining mucosal and systemic delivery of antigens from *Escherichia coli* [10] and HIV [11], for example. When *Neisseria* antigens are studied, OMVs from meningococci were good adjuvants for mixed delivery of *N. gonorrhoeae* antigens in mice [12]. Moreover, IN and systemic delivery of a meningococcal polysaccharide C conjugated to the diphtheria toxoid induced systemic bactericidal antibodies against *Neisseria meningitidis* and neutralizing antibodies against the toxoid [13].

However, not all adjuvants are suitable for mucosal and parenteral routes, thus supporting cellular and humoral responses. Dimethyldioctadecylammonium bromide (DDA), a cationic liposome, and Saponin (Sap), extracted from the bark of *Quillaja saponaria* Molina, can be used for mucosal and parenteral administration [14,15,16]. DDA has already been used in diverse studies, mainly in the cationic adjuvant formulation (CAF) 01, which comprises DDA and trehalose dibehenate [17]. Sap, as one component of ISCOM (Immunostimulating complex, an adjuvant formed by mixing cholesterol, phospholipids and Sap), was demonstrated to induce a potent immune response when complexed with diverse antigens [18].

Studying new adjuvants is extremely relevant. The more adjuvant options that are available, the easier it is for each country to develop and produce its vaccines, according to its financial and scientific-technological conditions. Thus, adjuvants can trigger specific arms of the immune response, thereby modulating an ideal immune response [19].

The present study investigated the humoral and cellular immune responses when OMV from *N. meningitidis* serogroup B, mixed with DDA or Sap, were administrated by intranasal (IN) prime and subcutaneous (SC) booster, thus comparing it with a standard adjuvant, aluminium hydroxide (AH).

## 2. Materials and Methods

### 2.1. OMV Extraction

The *N. meningitidis* strain B:4:nt was provided by the Bacteriology Center of Adolfo Lutz Institute (São Paulo, SP, Brazil). After several runs through culture medium, the strain did not express the polysaccharide capsule, as verified in Dot-ELISA using the monoclonal antibody 2-2-B, kindly provided by Dr Wendel Zollinger (data not shown). OMVs were obtained as previously described [20]. Briefly, the bacteria were grown in Tryptic soy broth (TSB) medium. Bacteria pellets were incubated with a 0.1 M sodium acetate and 0.2 M lithium chloride solution, and 2 mm glass beads, in a shaker, at 45 °C, for 2 h, at 300 rpm. It was then centrifuged and the supernatant was collected. Following extraction, OMVs were detoxified to reduce the lipopolysaccharide using Sepharose 4B column bound to polymyxin B, as described previously [21]. Residual LPS was determined using the QCL-1000 assay kit (Lonza, Walkerville, MD, USA), following the manufacturers’ instructions. The concentration was within the acceptable range for vaccines (<200 EU/mL) [22].

### 2.2. Adjuvants

#### Aluminium Hydroxide and DDA in Bilayer Fragments

The Aluminium hydroxide (AH) (Rehydragel) was prepared at a concentration of 0.1 mM and filtrated in a 0.45 µm filter membrane. DDA (Sigma-Aldrich, San Louis, MO, USA) was prepared by sonication method in a Sonics Vibra-Cell (Sonics and Materials, Newtown, CT, USA) at 25% amplitude for 20 min, forming bilayer fragments, subsequently centrifuged for 30 min, at 10.000 rpm and 4 °C, to remove ant titanium particles released by the macrotip, and filtered using a 0.45 µm filter membrane [21]. Both adjuvants, in the described concentrations, have been used before in our laboratory, proving its safety and efficacy with different *Neisseria* antigens [21,23,24,25].

### 2.3. Saponin and Hemolytic Analysis

The Sap preparation used the *Quillaja Saponaria* bark (Sigma-Aldrich, San Louis, MO, USA). Hemolytic analysis was performed to determine the Sap concentration when the adjuvant did not present toxicity for experimental use, as described previously [26]. In summary, a blood sample was collected, red blood cells were extracted, diluted in a concentration of 4% *v/v* in saline solution 0.1 M and combined with the different Sap concentrations; negative control (red blood cells only) and positive control (Triton x-100). Following this, the highest Sap concentration not demonstrating toxicity was diluted in saline solution 0.1 M and filtered through a 0.45 µm filter membrane.

### 2.4. Antigenic Preparations

All adjuvants were mixed with 25 µg/mL of OMV from *N. meningitidis* B:4:nt strain. The antigenic preparations were allowed to interact for one hour prior to immunization [27]. The OMV+DDA and OMV+Sap mixtures were characterized using the Zeta Potential Analyzer–Zeta PALS (Brookhaven Instruments, Holtsville, NY, USA. Size, charge and polydispersion were considered to choose the best adjuvant concentration.

### 2.5. Mice and Immunization

The 2-month-old female Swiss mice were obtained from animal facilities of Adolfo Lutz Institute, São Paulo, Brazil. All procedures involving animals met the Brazilian Code for Laboratory Animal Use guidelines and the study was approved by the Ethics Committee for Animal Use of the Adolfo Lutz Institute (number 01/2021). The Swiss lineage was used considering its similarity with human genetic background, as outbred mice [28]. In previous studies, we verified no differences between male and female mice, so we conducted the experiments with females [29].

A total of 30 animals were used in the study and divided into 5 groups: Control, OMV, OMV+AH, OMV+DDA, and OMV+Sap.

To verify DDA and Sap adjuvancity for mixed mucosal/parenteral immunization, a heterologous prime-booster scheme was applied: animals were immunized twice, 15 days apart. The prime consisted of 4 IN doses (0.25 µg of OMVs per dose) over 4 consecutive days; the booster was a single SC dose (2.5 µg of OMVs per dose) [21]. As the Sap group did not present statistically higher antibody titers after two doses, an extra SC booster was administrated. For antigen control, one group received OMV alone following the same IN/SC regimen and OMV concentration, while the non-immune control consisted of naïve animals maintained in the same conditions throughout the study.

Given that AH is an extensively used adjuvant [30], another group received OMVs adjuvanted with AH for comparison [31]. However, considering AH reactogenicity in the mucosa and the importance of comparing mucosal with systemic delivery [32], a homologous prime-booster approach was adopted, using the same immunization route for the whole immunization: two SC doses (2.5 µg of OMVs per dose), 15 days apart.

**Figure 1:**
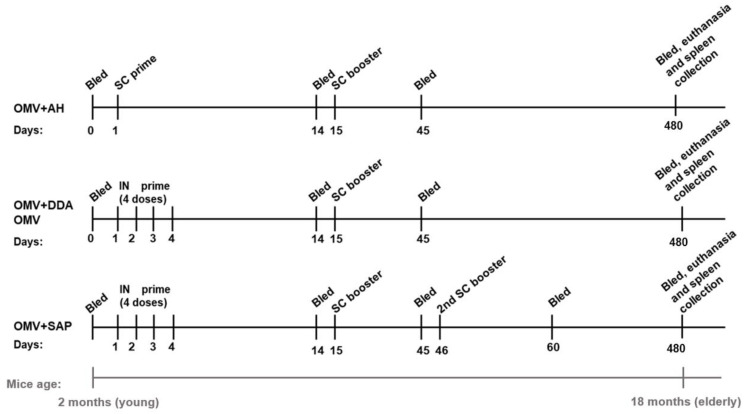
Immunization scheme and blood collections. AH: aluminium hydroxide; DDA: dimethyldioctadecylammonium bromide; IN: intranasal; OMV: outer membrane vesicles; Sap: Saponin; SC: subcutaneous.

Blood collection was performed by retro-orbital plexus puncture before immunization (pre-immune), 15 days after the prime and 30 days after the booster. When the mice completed 18 months, they were considered elderly [33]. Given the importance of studying the immune response of this age group [34], mice were bled to verify the persistence of the immune response and then, euthanized for spleen collection and the cellular memory was studied by ELISpot. Figure 1 shows the experimental calendar of the different immunization approaches.

### 2.6. Sodium Dodecyl-Sulfate-Polyacrylamide Gel Electrophoresis (SDS-PAGE)

OMVs were separated in a 10% polyacrylamide gel electrophoresis in a discontinuous system [35]. A molecular weight (MW) marker (New England Biolabs, Ipswich, MA, USA) was added in the electrophoretic run. After the electrophoresis, the gel was stained with Coomassie Blue (PhastGel Blue R, Amersham-Pharmacia Biotech, Amersham, UK).

### 2.7. Enzyme-Linked Immunosorbent Assay (ELISA) and Avidity Assay

High-binding polystyrene plates (Costar, Corning LifeSciences, Tewksbury, MA, USA) were coated with 5 µg/mL of *N. meningitidis* B:4:nt OMV, diluted in 0.1 M carbonate-bicarbonate buffer (pH 9.5), overnight at 4 °C. Plates were blocked with nonfat milk 5% (La Sereníssima, General Rodriguez, Buenos Aires, Argentina) for 2 h at 37 °C. The individual sera, diluted at 1:100, were incubated overnight, at 4 °C. The Horseradish peroxidase (HRP)-conjugated anti-mouse IgG (γ chain) (1:20,000), IgG2a or IgG3 (1:10,000) (Kirkegaard and Perry Laboratories, Gaithersburg, MD, USA) were incubated for 2 h, at 37 °C. For IgG1 analysis, the biotin-conjugated anti-mouse IgG1 (1:5000) (Kirkegaard and Perry Laboratories, Gaithersburg, MD, USA) and streptavidin-peroxidase (1:2000) (Zymed Laboratories, San Francisco, CA, USA) were used, being incubated for 1 h at 37 °C. The enzymatic reaction was developed with TMB (3,3′,5,5′-Tetramethylbenzidine) (Sigma-Aldrich, San Louis, MO, USA), for 20 min, at 37 °C. The reaction was stopped with H_2_SO_4_ 1 N and the optical densities (OD) were read at 450 nm in a microplate reader (Labsystem Multiskan, Thermo Fisher Scientific, Waltham, MA, USA).

The avidity index (AI) measures the binding strength between the antigens and the antibodies. It was determined by a modified ELISA, using an additional period of incubation with the chaotropic agent potassium thiocyanate (KSCN) 1.5 M, for 20 min at room temperature (RT) (20–25 °C), after sera incubation, followed by routine ELISA steps [36]. The AI was expressed as the ratio between the OD in the presence of KSCN/OD in the absence of KSCN and transformed into a percentage. The AI was classified as high if ≥50%, intermediate when between 30% and 49%, and low if <30% [37].

### 2.8. Serum-Bactericidal Assay (SBA)

The serum-bactericidal assay was conducted using the Tilt method [38]. Two-fold dilutions of pooled sera were used. The assay was conducted using pre-immune and immune sera collected when mice were young, after the 2 (OMVs, OMV+AH, OMV+DDA) or 3 doses (OMV+Sap) doses of immunization. Pooled sera samples were inactivated for 30 min at 56 °C. Briefly, the B:4:nt strain was cultured in TSB agar supplemented with 5% inactivated horse serum for 24 h. An isolated colony was suspended in Hank’s solution supplemented with 0.1% of glucose, 0.1% of bovine serum albumin and 9 mM of Hepes and set for OD_650nm_ = 0.1. The working suspension was diluted at a 1:500 ratio to yield approximately 50 colonies after incubation for 24 h, as determined previously in our laboratory. Diluted sera were mixed with guinea pig serum as an exogenous complement source [39] and working bacteria suspension on a sterile microplate. The microplate was incubated at 35 °C, 5% CO_2_, for 1 h. Afterward, 10 µL from each well was plaqued in TSB plates. The plates were incubated at 35 °C, 5% CO_2_, for 24 h and colonies were counted. The bactericidal titer was considered as the reciprocal dilution presenting ≥ 50% reduction of colonies compared with controls [38].

### 2.9. Immunoblot

SDS-PAGE was performed as described above. Following the separation, proteins were transferred to a 0.45 µm nitrocellulose membrane (Bio-Rad Laboratories, Hercules, CA, USA) in a transfer buffer (25 mM Tris, 192 mM Glycine, Methanol 20%, pH 8.3) over a period of approximately 24 h at 50 V. Strips containing approximately 10 µg of protein were blocked with nonfat milk 5% (La Sereníssima), overnight, at 4 °C. Afterwards, membranes were incubated for 2 h with pooled pre-immune or immune sera of mice collected after one (OMV, OMV+AH and OMV+DDA) or two (OMV+Sap) SC boosters, at a dilution of 1:50, at RT. Then, HRP-conjugated anti-mouse IgG (γ chain) (1:10,000) (Kirkegaard and Perry Laboratories, Gaithersburg, MD, USA) was incubated for 2 h, at RT. The enzymatic reaction was developed with 4-chloro-1-naphthol (Sigma-Aldrich, San Louis, MO, USA) and was stopped with distilled water after 20 min.

### 2.10. Enzyme-Linked Immunosorbent Spot (ELISpot) Assay

The ELISpot assay was conducted using individual spleens of mice. It was performed in 96-well polyvinylidene difluoride (PVDF) microtiter plates precoated with mAb anti-Interleukin (IL)-4 or IL-17 (Mabtech, Stockholm, Sweden). Plates were washed with sterile phosphate-buffered saline (PBS) and blocked with nonfat milk 5% (La Sereníssima, General Rodriguez, Buenos Aires, Argentina) at RT, for 2 h. Afterward, the plates were washed and spleen cells were added at 1 × 10^6^ cells/mL. The cells were stimulated with 50 µg/mL OMVs from strain B:4:nt, or 10 µg/mL of concanavalin A (ConA) (Sigma-Aldrich, San Louis, MO, USA) or added without stimulus. The plates were incubated for 16 h, at 37 °C, 5% CO_2_.

To remove the cells, plates were washed with PBS, then 100 µL/well of Ethylenediamine tetra acetic acid (EDTA) 1 mM was added and the plates were incubated for 10 min at 37 °C and washed again. Then, the biotinylated anti-IL-4 (1:1000) or anti-IL-17 (1:5000) (Mabtech, Stockholm, Sweden) was incubated for 2 h at RT. Following this, plates were incubated with streptavidin-alkaline phosphatase (ALP) (Mabtech, Stockholm, Sweden) diluted 1:1000, for 1 h, at RT. The enzymatic reaction was developed with the chromogenic substrate 5-bromo-4-chloro-3-indolyl-phosphate/nitro blue tetrazolium (BCIP/NBT-plus) (Mabtech, Stockholm, Sweden) until spots appeared. The reaction was stopped with distilled water. After drying, plates were read with the AID ELISpot Reader Version 7.0 (Autoimmun Diagnostika, Strassberg, Germany). The duplicate mean was calculated, and the results were expressed as IL-4 or IL-17 secretory cells per 10^6^ cells/mL, after removing the control wells with no stimuli.

### 2.11. Statistical Analysis

The statistical analysis was performed with the software GraphPad Prism 8 (GraphPad Software Inc., La Jolla, CA, USA). The results were submitted to the non-parametric Kruskal–Wallis test followed by Dunn’s post-hoc test for intergroup analysis, while the intragroup analysis used the non-parametric Friedman test, followed by Wilcoxon post-hoc test. *p* values ≤ 0.05 were considered significant.

## 3. Results

### 3.1. Electrophoretic Profile of OMVs

**Figure 2:**
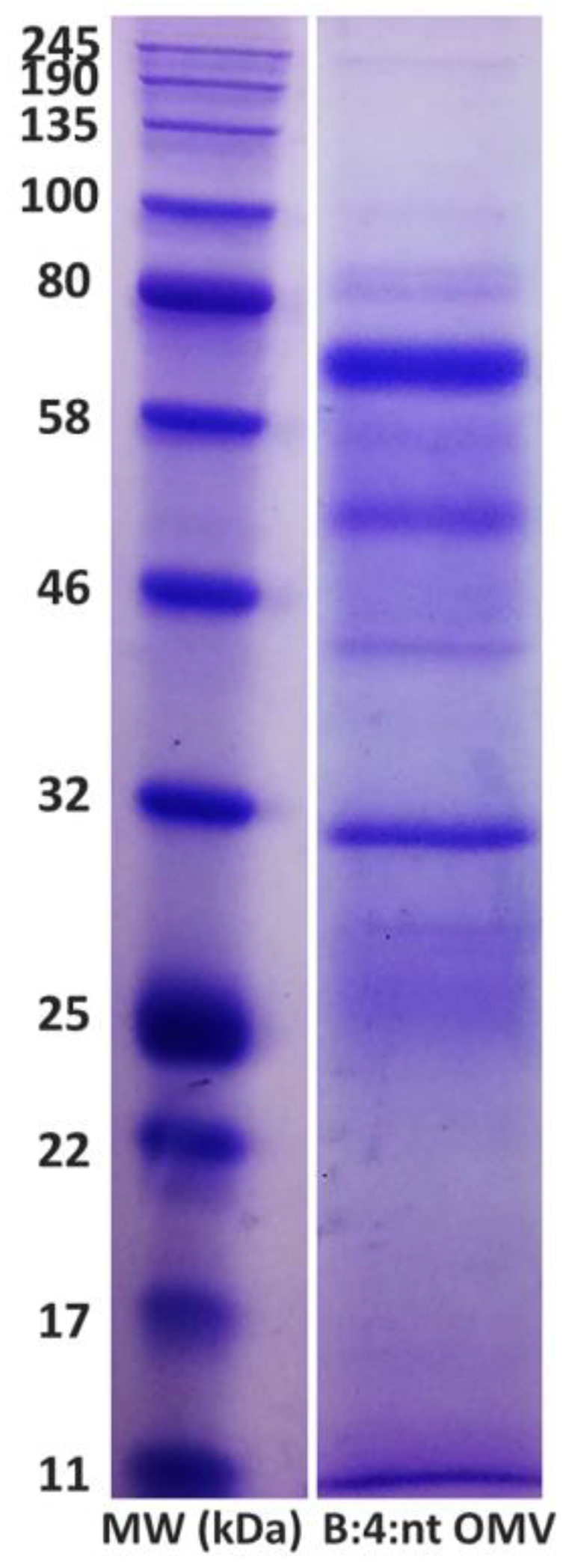
Electrophoretic profile of *N. meningitidis* Outer membrane vesicles (OMVs) from strain B:4:nt stained with Coomassie Blue. At the left, the molecular weight (MW) marker ranging from 11 to 245 kDa.

The electrophoretic profile of OMVs is shown in Figure 2. The OMVs expressed proteins in a range of 25 to 80 kDa.

### 3.2. Hemolytic Activity

The adjuvant *Quillaja Saponaria* can induce adverse events, such as systemic toxicity and hemolytic activity [40]. Given this, a hemolytic activity assay was conducted to identify a concentration where the adjuvant would not induce hemolysis, as shown in Table 1.

The concentrations within the range of 1 mg/mL to 50 µg/mL of *Quillaja saponaria* demonstrated high toxicity levels, but in concentrations below 10 µg/mL the extract demonstrated hemolysis lower than 5%, which is considered as low toxicity [26].

### 3.3. Zeta-Potential Analysis of Antigenic Preparations

The physical characteristics (size, charge and polydispersion) of the DDA and Sap adjuvants complexed to *N. meningitis* OMV were studied to verify which concentration presented better shape and stability. The results are in Table 2.

The concentration of DDA was maintained at 0.1 mM, given that this concentration was used before [21] and the OMV concentration varied (Table 2). The DDA complexed to OMVs shows a positive charge, being a cationic preparation. The size of the OMV+DDA nanoparticles varied from 116 to 206 nm and the polydispersion, from 0.309 to 0.339. Considering that an ideal size for lymphatic absorption is in the range of 40–200 nm [41] and an acceptable polydispersion value is 0.3 or less [42], the OMV concentration of 25 µg/mL was chosen for the experimental procedure, showing a polydispersion closest to 0.3 and size smaller than 200 nm.

The characterization of the Sap complexed to the OMVs was also conducted. Provided that the OMV+DDA preparation would use 25 µg/mL of OMVs, this concentration was fixed, while Sap concentration varied (Table 3).

The Sap concentration of 50 µg/mL presented a smaller size, but the same dose demonstrated a high rate of hemolysis (Table 1). Therefore, the Sap concentration of 10 µg/mL was chosen to perform the immunization, since this concentration was smaller than 260 nm when mixed with OMVs (Table 3).

### 3.4. Antibody Levels Evaluation

**Figure 3:**
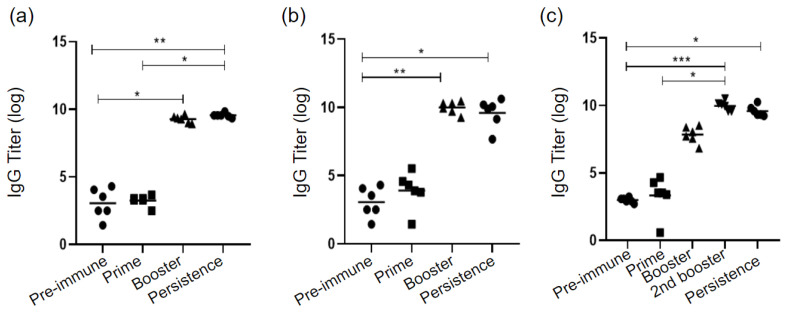
Analysis of IgG titers during the experimental period, by ELISA, in groups (**a**) OMV+AH, (**b**) OMV+DDA and (**c**) OMV+Sap. For statistical analyses, it was used the Friedman test followed by Wilcoxon post-hoc test. * *p* < 0.05; ** *p* < 0.01; *** *p* < 0.001; AH: aluminium hydroxide; DDA: dimethyldioctadecylammonium bromide; OMV: outer membrane vesicles; Sap: Saponin.

Figure 3 shows the IgG titers measured, by ELISA, in mice sera before and after each immunization dose. No group, regardless of the adjuvant used, was able to induce a significant IgG production after the prime. The OMV+AH and OMV+DDA groups demonstrated an increase in antibody production after the booster. However, Sap required an extra SC to finally increase IgG titers. All groups presented IgG titers significantly higher than controls when mice were 18 months old. The OMV group did not present statistically higher titers than controls, therefore, this group is not represented in the image.

**Figure 4:**
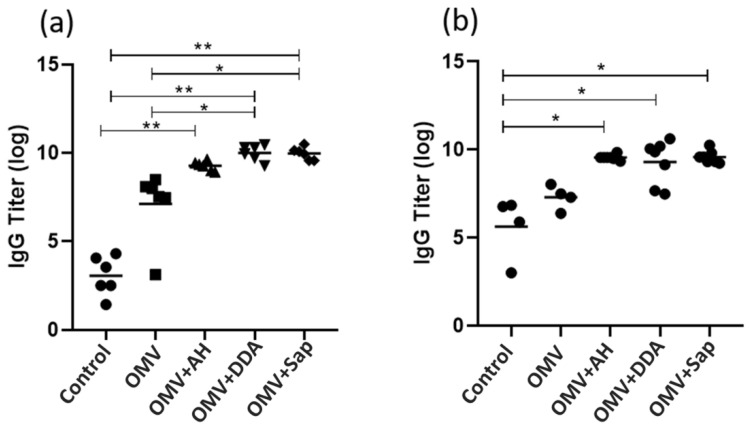
Antibody titer analysis of (**a**) Total IgG 30 days after the subcutaneous booster (OMV, OMV+AH and OMV+DDA) or 15 days after the 2nd booster (OMV+Sap); (**b**) persistence of total IgG, when mice were 18 months old. * *p* < 0.05; ** *p* < 0.01; AH: aluminium hydroxide; DDA: dimethyldioctadecylammonium bromide; OMV: outer membrane vesicles; Sap: Saponin.

Since IgG titers were statistically higher than pre-immune levels only after the whole immunization schedule, the following analysis focused on samples collected after the whole immunization (two doses for OMV, OMV+AH and OMV+DDA and three doses for OMV+Sap). An intergroup analysis of IgG titers was conducted in samples collected when mice were young (Figure 4a) and when mice were elderly (Figure 4b). The OMV+AH, OMV+DDA and OMV+Sap groups demonstrated a similar increased IgG production, whereas the OMV group, without adjuvant, showed no increase, demonstrating the importance of the adjuvants (Figure 4a). Moreover, antibody levels were maintained even when the animals were older (Figure 4b). When the adjuvants were compared to each other, we could not verify superiority of IgG induction. Although, OMV+AH was only superior to the control, while OMV+DDA and OMV+Sap were superior to the control and the OMV alone (Figure 4a).

**Figure 5:**
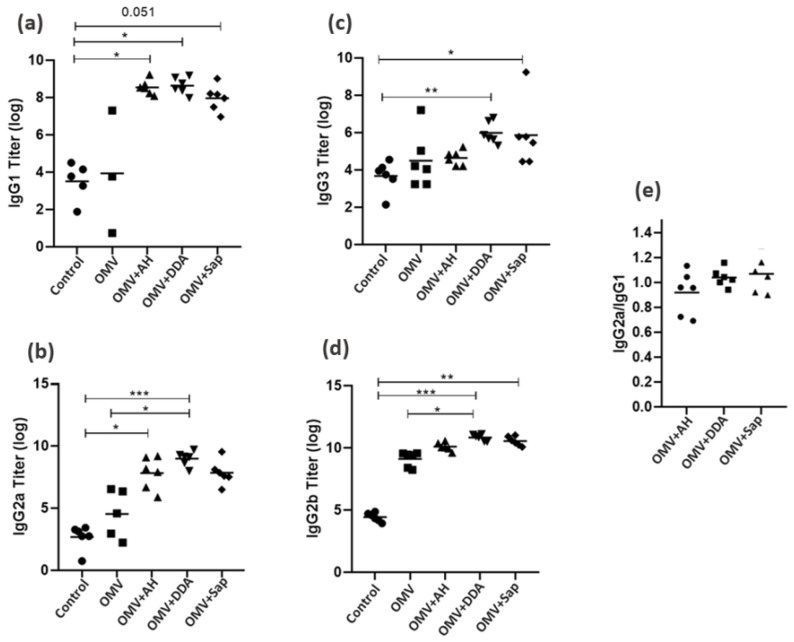
(**a**) IgG1; (**b**) IgG2a; (**c**) IgG23; (**d**) IgG2b levels in sera of young mice. (**e**) IgG2a/IgG1 ratio after the whole immunization schedule. For statistical analysis, we used the Kruskal–Wallis Test, followed by Dunn’s post-hoc test. Dots: control; black squares: OMV; trangle: OMV+AH; inverted trangle: OMV+DDA; rhombus: OMV+Sap; * *p* < 0.05; ** *p* < 0.01; *** *p* < 0.001; AH: aluminium hydroxide; DDA: dimethyldioctadecylammonium bromide; OMV: outer membrane vesicles; Sap: Saponin.

The IgG isotypes and the IgG2a/IgG1 ratio were also assessed (Figure 5). The OMV+DDA group showed increased IgG1, IgG2a, IgG2b and IgG3 levels; the OMV+AH group presented increased IgG1 and IgG2a levels, and the OMV+Sap group showed higher IgG2b and IgG3 levels. Finally, all adjuvanted groups demonstrated a balanced IgG1/IgG2a profile.

### 3.5. Antibodies Functionality Evaluation

The avidity index (AI) is an important parameter to monitor humoral response, so the IgG avidity of the OMV+AH, OMV+DDA, OMV+Sap and OMV groups was analyzed (Table 4). All groups demonstrated an intermediate IgG avidity (Table 4), with a similar binding strength.

The serum bactericidal activity (SBA) is the main tool to determine the immunogenicity of meningococcal vaccines, once it evaluates the ability of the immune serum, along with an exogenous complement, to kill *N. meningitidis* in vitro [43]. Table 4 also presents the SBA results. The OMV, without adjuvants, led to bactericidal activity (1/4 titer). However, the OMV+AH, OMV+DDA and OMV+Sap groups presented a bactericidal titer 8× higher (1/32 titer), proving that adjuvants supported the functionality of the antibodies.

### 3.6. Antigenic Recognition Evaluation

**Figure 6:**
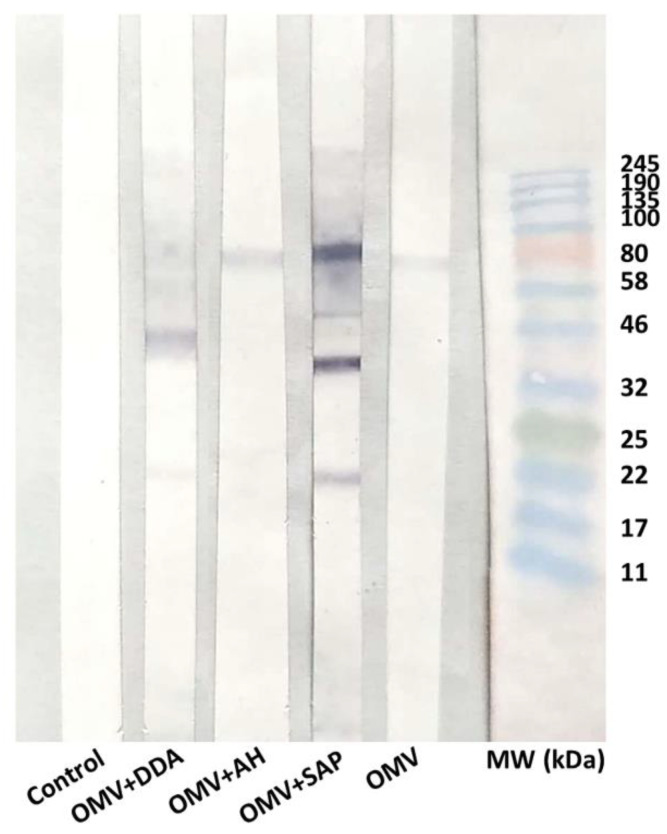
Evaluation of the antigenic recognition by Immunoblot. Sera were collected when mice were young—before the immunization (Control), after two (OMV+AH, OMV+DDA OMV) or three (OMV+Sap) doses of immunization. AH: aluminium hydroxide; DDA: dimethyldioctadecylammonium bromide; MW: molecular weight; OMV: outer membrane vesicles; Sap: Saponin.

The recognition of proteins present in *N. meningitidis* B:4:nt strain by Immunoblot were evaluated. The pooled sera of mice, after the last dose of immunization was used. The sera of the OMV+Sap group showed higher reactivity to the antigens, with proteins of approximately 22, 46, 58 and 80 kDa, whereas the OMV and OMV+AH groups recognized an 80 kDa protein; while the OMV+DDA group recognized a 46 kDa protein (Figure 6). The molecular weight of the bands suggests that the antigens recognized were Tbp (80 kDa), PorA (46 kDa) and Opa (25 kDa) [44].

### 3.7. Cytokine Evaluation

**Figure 7:**
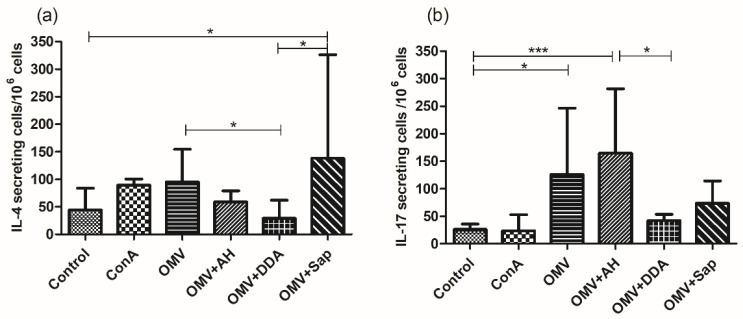
Cytokine analysis by ELISpot, when mice were 18 months old. The production of (**a**) IL-4 and (**b**) IL-17 by splenocytes were compared between the groups. For statistical analysis, it was used the Kruskal–Wallis test followed by Dunn’s post-hoc test. * *p* < 0.05 and *** *p* < 0.001. AH: aluminium hydroxide; DDA: dimethyldioctadecylammonium bromide; OMV: outer membrane vesicles; Sap: Saponin.

The CD4+ cells secrete cytokines, which augments IgG production and isotype differentiation [45]. In the experimental model, the Th1 cytokine INF-γ mostly induces IgG2a and IgG2c, whereas the Th2 cytokine IL-4 induces IgG1 and IgG2b [46]. It is well known that a Th2 pattern, which supports antibody production, is the ideal mechanism to protect against *N. meningitidis*, inducing bactericidal antibodies [44]. The OMV+Sap group presented an increase in IL-4 in relation to the OMV+DDA and Control groups, while the OMV group presented an increase in IL-4 in comparison to the OMV+DDA group (Figure 7a).

Considering that *N. meningitidis* is an extracellular pathogen, the immune response against it can be supported by IL-17 secretion, a hallmark of Th17 response, so the induction of IL-17 was also verified (Figure 7b) [47]. The OMV+AH group presented an increase in IL-17 production when compared to the Control and OMV+DDA groups, and the OMV group demonstrated an increase of this cytokine in comparison to the Control group.

Due to mice age, by the time the ELISpot assay was carried out, part of the animals of each group had died. This might imply a low statistical power during analysis.

## 4. Discussion

Mucosal delivery is a promising field in vaccine development, given that it induces local immunity and supports cellular responses. Therefore, it is important to study adjuvants adequate for this route [48,49]. The present study evaluated the immune response triggered by three different antigenic formulations, containing three different adjuvants (AH, DDA and Sap) complexed with *N. meningitidis* OMVs. While DDA and Sap are proposed for mixed mucosal/parenteral immunization, AH is the standard used to compare both adjuvant properties [31] and systemic immunization routes [32].

Firstly, the physical characteristics of antigenic preparations (OMVs mixed with DDA or Sap) were evaluated. A safe, stable and efficient formulation should present homogenous size, with a polydispersion index below 0.3 [50,51]. The concentration of DDA and Sap used in the experimental study did not present a high level of homogeneity (Table 2 and Table 3), implying a low stability formulation. The addition of cholesterol to the formulation is a good alternative to improve stability and decrease particle size [52], which could make it a good alternative to improve the formulations. Both preparations presented a size smaller than 260 nm, suitable for uptake by antigen-presenting cells (Table 3) [53].

The presence of IgG is important to combat the pathogen. One common characteristic, observed in all groups, is that the prime dose alone could not induce significant IgG production (Figure 3). This result agrees with the literature, which describes the importance of booster doses [12,21].

However, after the booster, IgG titer increased in the OMV+AH and OMV+DDA groups, whereas the OMV+Sap group required an additional dose to achieve a significant increase in IgG titers (Figure 3). Another relevant point is that these three groups maintained the IgG titer even when the mice were 18 months, which is considered old [54]. It is desirable for vaccines to induce persistent antibodies [55]. Nanoparticulated and aluminium-based adjuvants also contributed to the persistence of the humoral response to *Bacilus anthracis* [56] and Influenza [57]. Immune system functionality is affected during ageing, with reduced B and T cells and a decreased antibody response usually being observed. Therefore, the use of adjuvants that support the immunologic memory for longer periods shows promise [58].

In general, the IgG2a/IgG1 ratios of the three adjuvants were close to 1, suggesting a mixed Th1/Th2 profile of response (Figure 5). Although, most individuals from the OMV+AH group showed a Th2 tendency, which agrees with the literature [59]; while DDA and Sap mean values point towards to Th1, also expected according to published studies [60,61].

Of note, it is interesting to verify the isotypes according to functionality of antibodies, given that the main correlate of protection from meningococcal disease is bactericidal antibodies [44]. In vitro studies demonstrated that IgG3 is an effective activator of the complement system via the classic pathway, which is important to achieve a higher vaccine efficacy against extracellular pathogens, such as *N. meningitidis* [62]. Interestingly, the OMV+DDA and OMV+Sap were the only groups to show increased IgG3 (Figure 5). Hence, a study verified that the four murine IgG isotypes differ in bactericidal activity, showing that it respected the following classification: IgG3 > IgG2b > IgG2a > IgG1 [63]. Therefore, Sap and DDA would modulate the response to the best isotypes for bactericidal activity, followed by AH and, then, OMVs without adjuvants.

As functional parameters, the IgG avidity and the SBA were evaluated. Increasing avidity of the antibodies reflects the affinity maturation process and is important to predict vaccine efficacy [64,65,66]. Hence, increased avidity has been correlated with bactericidal activity [67]. As seen in Table 4, all groups presented intermediate avidity, although OMV+AH avidity was slightly higher. This was found in previous studies [24,25].

SBA has been adopted as a “gold standard” test for immunity against meningococcal disease [66]. SBA titers ≥ 1/4 are considered protective in humans and widely accepted as a surrogate of vaccine efficacy [68]. All groups showed bactericidal titers (Table 4). Even though the avidity indexes of the antibodies were only intermediate, the increased titers might have efficiently opsonized the bacteria for complement-mediated lysis [67]. OMV alone induced a bactericidal titer of 1/4, which agrees with the literature about OMV immunogenicity [69]. However, the adjuvants improved the bactericidal activity, increasing the bactericidal titers, and thereby proving their importance.

It is also important to address which antigens were recognized by immune sera. OMV and OMV+AH groups recognized one antigen in the molecular range of Tbp, which is interesting, given that it is a conserved protein, capable of inducing bactericidal antibodies even at a low concentration [70,71,72]. OMV+DDA recognized PorA, which is highly heterogeneous among meningococcal strains, but it is effective to induce bactericidal antibodies [44]. OMV+Sap recognized more proteins, possibly Tpb, PorA and Opa, the latter is heterogeneous but important for the immune response to meningococci [20]. Even though all groups presented bactericidal activity, the recognition of more antigens by the OMV+DDA and OMV+Sap groups would possibly increase the chances of this group presenting cross-reaction if different strains were investigated [73].

When all these parameters are taken together, the elevated IgG titers, the presence of IgG2b and IgG3 isotypes and recognition of multiple antigens corroborate the SBA titers of OMV+DDA and OMV+Sap groups. On the other hand, the bactericidal activity presented by OMV and OMV+AH is yet to be explored. While the OMV group presented a low SBA titer, OMV+AH was comparable to the other adjuvants. We hypothesized that the antigen presentation might have played a role in these results. While ELISA presents the antigen in the native form, similarly to the bacteria suspension in SBA, the Immunoblotting protocol used denatures the antigens [35]. In other studies, we verified that OMV+AH supports multiple antigenic recognition [24,25] and, in here, ELISA pointed to increased IgG titers. A further analysis using Empigen BB^®^ in Immunoblotting to increase the Porin A and Porin B epitopes exposition, could address this issue [74], or another Immunoblotting could be carried out not using denaturing conditions.

The cytokine secretion is another important parameter to consider. Cytokines orchestrates the immune response: IL-4 is known to support the humoral response and IL-17 supports the immunity against extracellular pathogens and in the mucosa [44,47]. Thus, mice were immunized when young and ELISpot was carried out when mice were elderly [33], so we could verify the immunologic memory. Considering the ageing of the population and the increasing incidence of meningococcal disease in the elderly, it is relevant to verify this parameter [34].

While ELISA results could not point to the superiority of the adjuvants compared, ELISpot results (Figure 7) suggest that Sap and AH contributed more than DDA to cytokine release. But, considering that the assay was carried out when mice were elderly, it would be more adequate to state that Sap and AH would have provided better immunologic memory. The literature suggests that AH mechanisms induce immunological memory cells [75], as well as a Sap-based adjuvant [76]. DDA is also known to improve immune memory, thus, antibody titers were maintained. One possibility for the apparent failure in this study might be the cytokines analyzed. Given that this adjuvant supports a Th1 response [60], we can hypothesize that IFN-γ or IL-1 cytokines would have been detected if tested.

Sap is known to induce a Th1 response [61], however, we found only a slightly increase of IgG2a/IgG1 ratio and robust IL-4 secretion. This might suggest at least a mixed Th1/Th2 response, what has been reported before [77]. OMV+AH presented high IgG1 levels and secreted IL-4; features of a Th2 pattern, as observed for AH [59]. Interestingly, this group secreted the higher IL-17 levels as well. A Th17 response induces protection mediated by neutrophil recruitment, inducing the liberation of antimicrobial peptides, thus triggering a Th1 response [78], and the IgG2a isotype, a feature of a Th1 response, was observed in the OMV+AH group. The possibility of a mixed response for OMV+Sap and OMV+DDA is interesting, since it was beneficial in some meningococcal vaccines [79].

The delivery of OMVs alone induced memory cells, as splenocytes of this group released both IL-4 and IL-17. Even though this group did not present other important immunological parameters discussed so far (IgG titers and isotypes, antigenic recognition and bactericidal activity), this result shows the adequacy of OMVs as a meningococcal vaccine antigen, eliciting a Th2 response, the hallmark of meningococcal protection [44] and Th17, a promising arm of the immune response as well [47].

## 5. Conclusions

In summary, OMVs were immunogenic, but adjuvants improved the formulations in several aspects. The three adjuvants (AH, DDA and Sap) induced higher bactericidal titer (1/32 versus 1/4 titer of OMVs alone), which is the main correlate of protection against meningococcal disease and contributed to persistent IgG titers and intermediate avidity. AH showed the higher avidity index and robust IL-17 release, but it failed to elicit all IgG isotypes. DDA supported antigenic recognition and all isotype differentiations, but seemed to induce poor immunological memory through IL-4 or IL-17 release. Sap was found to be a promising adjuvant option for *N. meningitidis* OMVs. Even though it needed one extra booster for the antigenic concentration used, it induced all IgG isotypes, immunological memory and multiple antigenic recognition.

## Figures and Tables

**Table 1:** Hemolytic activity of different concentrations of *Quillaja saponaria* extract.

	Hemolysis %						
[ ] Sap	1 mg	500 µg	100 µg	50 µg	10 µg	5 µg	1 µg
Sample 1	109.61	100.71	103.04	113.80	3.93	2.45	2.93
Sample 2	96.28	102.78	103.84	96.28	3.64	2.42	2.99
Sample 3	94.32	92.17	100.12	98.40	3.49	2.22	2.69
Mean	100.07	98.55	102.34	102.83	3.69	2.36	2.87

Sap: Saponin.

**Table 2:** Physical properties of DDA complexed to different concentrations of *N. meningitidis* OMVs.

[ ] DDA	[ ] OMV	Size (nm)	Polydispersion	Charge (mV)
0.1 mM	5 µg/mL	118.1 ± 4.9	0.333 ± 0.006	15.42 ± 1.78
0.1 mM	10 µg/mL	116.9 ± 4.8	0.324 ± 0.007	18.51 ± 1.24
0.1 mM	25 µg/mL	131.6 ± 4.6	0.309 ± 0.006	8.49 ± 2.18
0.1 mM	50 µg/mL	202.3 ± 4.8	0.339 ± 0.21	12.01 ± 0.72

DDA: dimethyldioctadecylammonium bromide; OMV: outer membrane vesicles.

**Table 3:** Physical properties of Sap complexed to *N. meningitidis* OMVs.

[ ] Sap	[ ] OMV	Size (nm)	Polydispersion	Charge (mV)
50 µg/mL	25 µg/mL	218.7 ± 12.1	0.359 ± 0.015	−13.64 ± 1.51
10 µg/mL	25 µg/mL	255.5 ± 17.9	0.383 ± 0.021	−10.5 ± 3.52
5 µg/mL	25 µg/mL	472.5 ± 52.7	0.359 ± 0.018	−22.78 ± 0.7

OMV: outer membrane vesicles; Sap: Saponin.

**Table 4:** Functional parameters of antibodies in sera of young mice, collected after the whole immunization schedule.

Functionality of Antibodies		
	Avidity Index (%)	Bactericidal Titer
OMV+AH	44.67	1/32
OMV+DDA	42.38	1/32
OMV+Sap	39.24	1/32
OMV	37.15	1/4

The avidity index refers to the mean value of each group and bactericidal titer refers to pooled sera of each group. AH: aluminium hydroxide; DDA: dimethyldioctadecylammonium bromide; OMV: outer membrane vesicles; Sap: Saponin.

## Data Availability

Original data is available upon request to the correspondent author.
